# Unilateral Arm Swelling on Apixaban: Central Venous Thrombosis Missed by Point-of-Care Ultrasound

**DOI:** 10.7759/cureus.109967

**Published:** 2026-05-31

**Authors:** Ryan Magee, Patrick Reese, Cody Due

**Affiliations:** 1 Department of Emergency Medicine, University of Oklahoma Health Sciences Center, Tulsa, USA

**Keywords:** catheter-associated thrombosis, central venous catheter thrombosis, central venous thrombosis, gi cancer, point-of-care ultrasound, upper extremity deep venous thrombosis

## Abstract

A 57-year-old man with metastatic colorectal cancer, a left internal jugular port, and reported apixaban use presented to the emergency department with worsening unilateral left upper-extremity swelling. Overnight point-of-care ultrasound did not identify a definite deep venous thrombosis (DVT), and he was discharged with next-morning formal imaging because formal vascular ultrasound was unavailable overnight. Formal imaging later that same day showed axillary thrombosis, and contrast CT of the neck and chest revealed bilateral internal jugular thrombosis, chronic left brachiocephalic thrombosis, suspected acute-on-chronic central venous thrombosis of the left upper extremity, and possible extension into the right transverse sinus. Venography and intravascular ultrasound later demonstrated near-occlusive thrombus involving the left axillary, subclavian, and brachiocephalic veins with severe underlying brachiocephalic stenosis. Aspiration thrombectomy and venoplasty improved central venous outflow. This case illustrates that persistent unilateral arm swelling in a high-risk oncology patient may reflect central venous thrombosis rather than isolated peripheral upper-extremity DVT. A negative peripheral or bedside ultrasound should not close the workup when symptoms and risk factors remain concerning for central disease.

## Introduction

Unilateral arm swelling is a common emergency department complaint and is often evaluated for upper-extremity deep venous thrombosis (DVT). In patients with active malignancy, an indwelling port, or prior central venous instrumentation that has since been removed, the differential must extend beyond isolated peripheral thrombosis because catheter-associated thrombosis may involve peripheral veins, central veins, or both [1-5]. This anatomic limitation is clinically important. Bedside or standard upper-extremity ultrasound can evaluate many compressible peripheral venous segments, but it may not adequately assess the subclavian vein, brachiocephalic veins, or superior vena cava [2,6]. Central venous thrombosis or stenosis can therefore remain occult when a peripheral or point-of-care study is negative. We report this case because the initial bedside ultrasound did not identify definite thrombosis, yet subsequent imaging revealed multifocal central venous thrombosis with severe brachiocephalic stenosis. The case highlights a practical diagnostic pitfall for emergency clinicians: in high-risk patients with persistent swelling, a negative peripheral study must be interpreted within the patient’s risk profile and the anatomy that the study can and cannot evaluate.

## Case presentation

A 57-year-old man with metastatic, moderately differentiated adenocarcinoma of the transverse colon and pulmonary metastasis presented during an overnight emergency department visit with worsening left upper extremity swelling. His history included prior right internal jugular and right-sided port-associated thrombosis, removal of that port approximately two years earlier, and later placement of a left internal jugular port. He was taking apixaban 5 mg twice daily with reported adherence, although he reported missing one dose approximately three weeks earlier. He denied chest pain, shortness of breath, nausea, vomiting, acute visual change, focal neurologic deficits, and seizures.

Initial concerns included upper-extremity DVT, catheter-associated thrombosis, more extensive central venous thrombosis or obstruction, pulmonary embolism, malignant venous compression, cellulitis, and lymphedema. Vital signs during the initial evaluation were temperature 36.6 °C, heart rate 97 beats/minute, respiratory rate 18 breaths/minute, blood pressure 141/65 mmHg, and oxygen saturation 100% on room air. Physical examination documented left upper-extremity swelling and bilateral lower-extremity swelling. He had no documented respiratory distress and was alert and oriented. The record did not further characterize the left arm swelling by degree, pitting, warmth, erythema, hand involvement, or visible collateral chest wall veins.

An overnight bedside or point-of-care ultrasound did not identify a definite DVT. Formal vascular imaging was unavailable overnight, and he was discharged for next-morning imaging. The formal study later that same calendar day showed thrombosis in the mid to proximal axillary vein, and he returned for admission that day. Initial laboratory testing showed leukopenia, anemia, thrombocytopenia, mild hyponatremia, and hypokalemia with preserved renal function. These abnormalities were interpreted in the context of active metastatic malignancy and ongoing systemic therapy rather than as direct causes of thrombosis (Table 1).

**Table 1:** Initial laboratory findings on admission WBC, white blood cell count

Laboratory Test	Result	Reference Range	Interpretation
WBC	3.3 × 10³/µL	4.5-11.0 × 10³/µL	Low
Hemoglobin	9.0 g/dL	13.5-17.5 g/dL (male)	Low
Platelets	127 × 10³/µL	150-400 × 10³/µL	Low
Sodium	132 mmol/L	136-145 mmol/L	Low
Potassium	3.2 mmol/L	3.5-5.0 mmol/L	Low
Creatinine	0.91 mg/dL	0.6-1.2 mg/dL	Normal

Contrast CT of the neck and chest performed after the positive formal ultrasound demonstrated an acute-appearing expansile thrombosis of the right internal jugular vein with possible extension into the right transverse sinus. MR venography or CT venography of the head was recommended. The same study showed chronic thrombosis of the lower left internal jugular vein and left brachiocephalic vein, with suspected acute-on-chronic thrombosis of the left upper-extremity venous vasculature and associated left arm, axillary, and chest wall swelling. These findings shifted the working diagnosis from isolated peripheral upper-extremity thrombosis to multifocal central venous thrombosis.

He was admitted to an inpatient telemetry unit, oncology was consulted, apixaban was withheld, and anticoagulation was changed to parenteral therapy, initially with argatroban and later with heparin infusion. The CT report also raised concern for extension into the right transverse sinus, but confirmatory MR venography was not documented. Inpatient notes did not describe neurologic compromise or respiratory instability.

**Figure 1:**
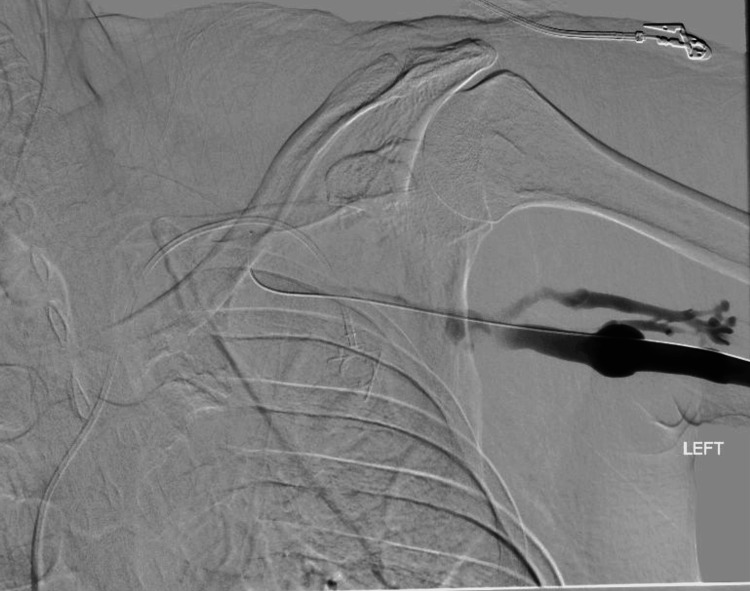
Pre-intervention left upper-extremity venography demonstrating near-occlusive thrombus involving the left axillary/subclavian venous outflow with poor central drainage

**Figure 2:**
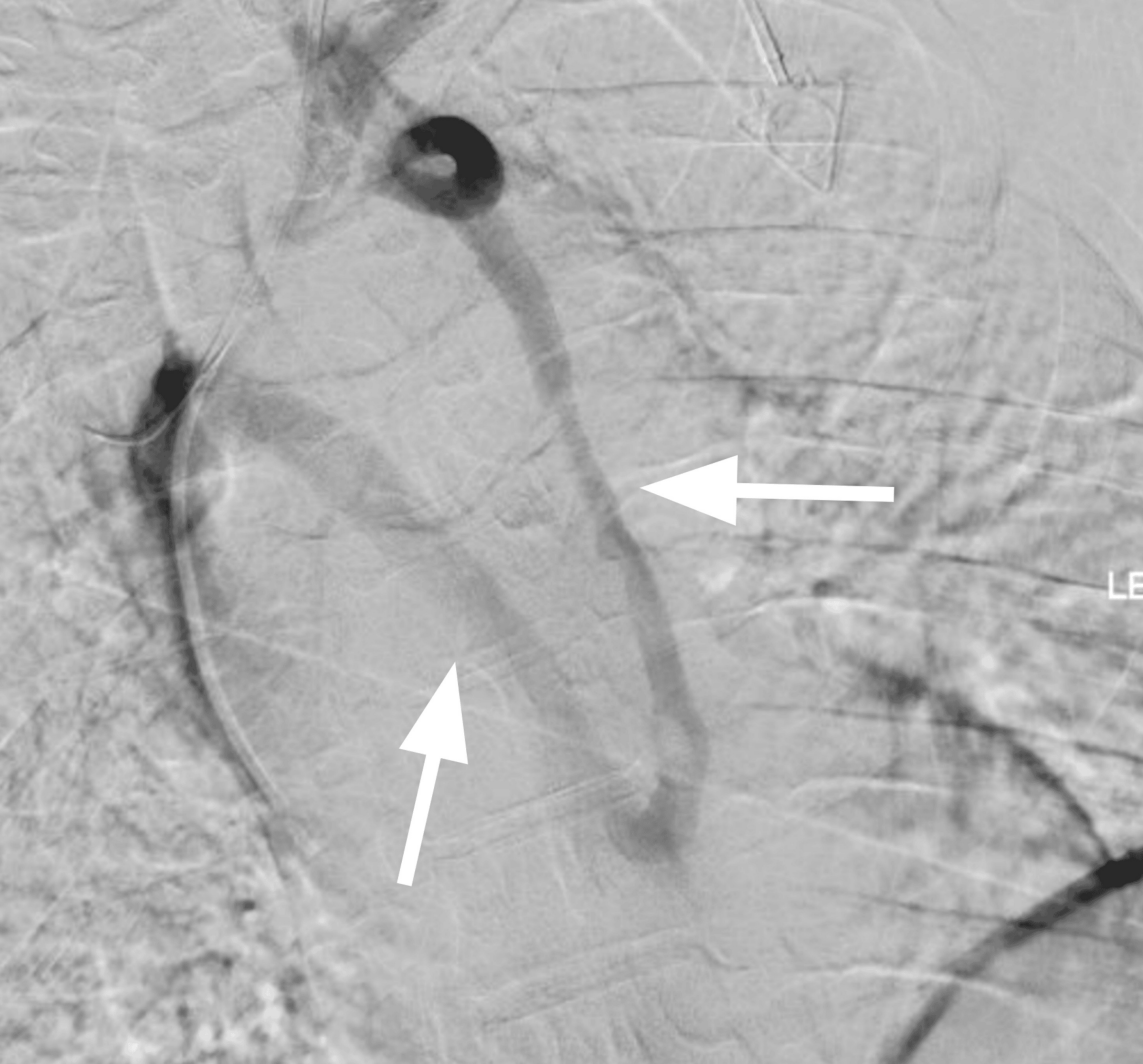
Venography demonstrating narrowing of the left brachiocephalic vein with collateral mediastinal venous drainage into the azygos system The arrows identify collateral venous pathways developed around the obstructed central venous segment.

**Figure 3:**
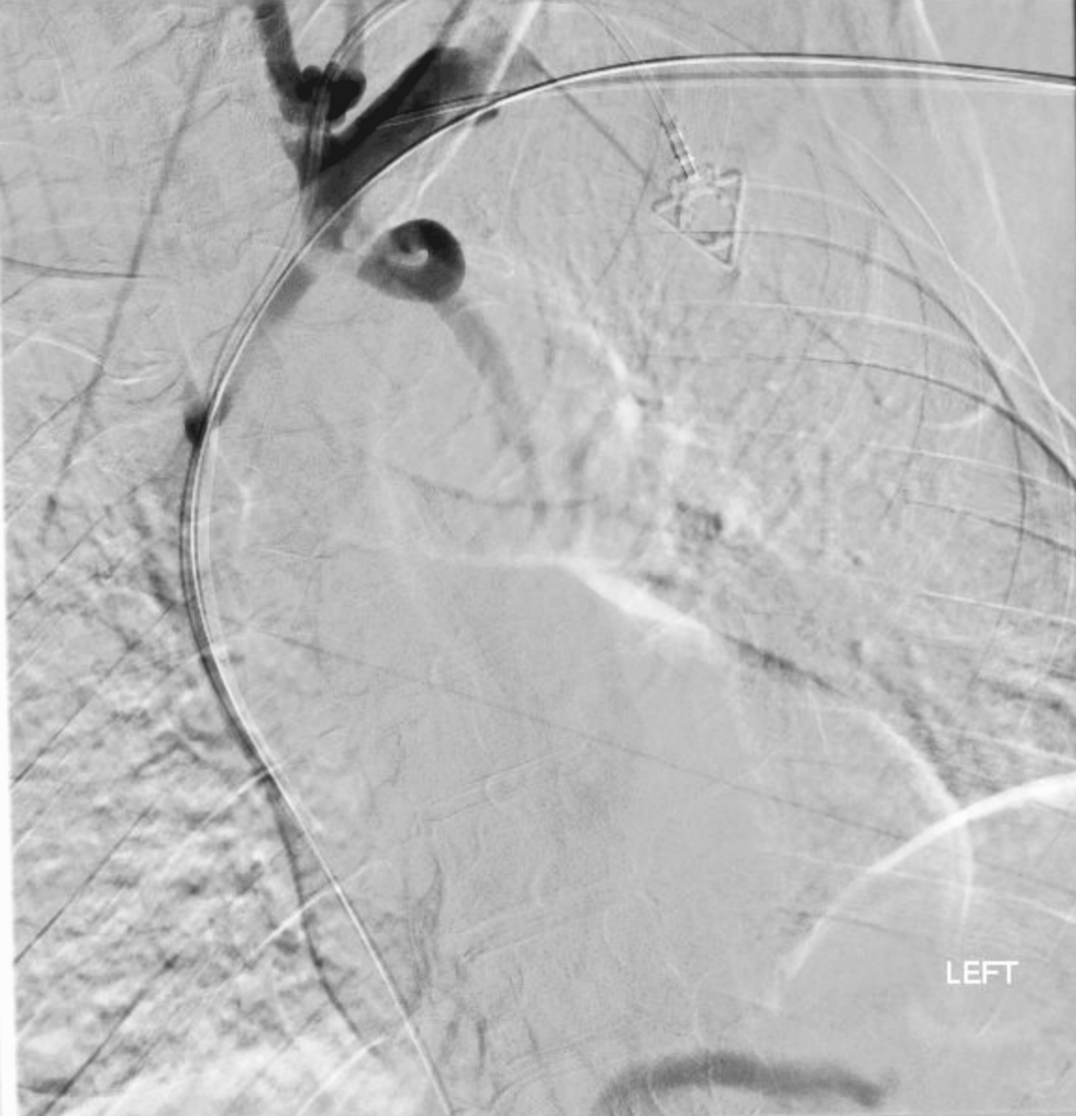
Post-intervention venography after aspiration thrombectomy and venoplasty demonstrating improved patency and central venous outflow through the left brachiocephalic/superior vena cava pathway, with residual small-caliber central venous disease

Repeat CT venography on hospital day six showed bilateral internal jugular thrombosis and again suggested a mixed acute-on-chronic process. On hospital day seven, interventional radiology performed left upper-extremity and central venography with intravascular ultrasound, aspiration thrombectomy, and central venoplasty. Venography and intravascular ultrasound demonstrated occlusive to nearly occlusive thrombus involving the left axillary, subclavian, and brachiocephalic veins, with severe underlying left brachiocephalic stenosis (Figure 1). The superior vena cava was patent, and collateral venous pathways were present (Figure 2). Aspiration thrombectomy and venoplasty were performed and improved flow, although residual small-caliber central venous disease remained (Figure 3). The port was left in place because it remained necessary for oncologic treatment. Later progress notes demonstrated gradual pain improvement with persistent edema, but no headache, dyspnea, chest pain, focal weakness, or seizures.

The key chronological events are summarized in Table 2.

**Table 2:** Clinical timeline of presentation, imaging, and management ED, emergency department; CT, computed tomography

Time point	Event	Diagnostic or management significance
Initial overnight ED visit	Presented with worsening left upper-extremity swelling while taking apixaban; no documented respiratory distress or neurological deficit.	High-risk presentation because of metastatic cancer, an indwelling left internal jugular port, and prior port-associated thrombosis.
Initial overnight ED evaluation	A bedside or point-of-care ultrasound did not identify definite deep venous thrombosis; formal vascular imaging was unavailable overnight.	Peripheral bedside imaging did not explain persistent swelling in a high-risk patient.
Next morning/same calendar day	Formal imaging showed thrombosis in the mid to proximal axillary vein; the patient returned and was admitted.	Diagnosis changed from a negative bedside study to confirmed thrombosis requiring inpatient management.
After positive formal imaging	Contrast CT neck and chest showed bilateral internal jugular thrombosis, chronic left brachiocephalic thrombosis, and suspected acute-on-chronic central venous thrombosis.	Raised concern for central venous thrombosis rather than isolated peripheral upper-extremity deep venous thrombosis.
Hospital day six	Repeat CT venography suggested bilateral internal jugular thrombosis and mixed acute-on-chronic disease.	Supported persistent multifocal central venous disease.
Hospital day seven	Venography with intravascular ultrasound showed near-occlusive thrombus in the left axillary, subclavian, and brachiocephalic veins with severe left brachiocephalic stenosis; aspiration thrombectomy and venoplasty improved flow.	Confirmed central venous disease and treated thrombotic obstruction plus underlying stenosis.

## Discussion

This case illustrates a common emergency department problem with an uncommon anatomic explanation. In a patient with malignancy, prior catheter thrombosis, and an indwelling port, unilateral arm swelling raises concern for more than routine peripheral upper-extremity DVT [1-5]. The later CT and venographic findings demonstrated that the patient’s presentation was driven by multifocal central venous thrombosis with severe left brachiocephalic stenosis rather than a simple isolated peripheral clot.

Guideline-based imaging principles not only support ultrasound as the usual first-line test for suspected upper-extremity DVT, but also recognize the limitations of ultrasound for thoracic venous segments and the role of CT venography, MR venography, or catheter venography when central venous disease is suspected, or ultrasound is nondiagnostic [2,6]. In this case, the overnight point-of-care study did not identify definite thrombosis, but it did not fully resolve the clinical question because the patient’s risk profile and persistent swelling remained concerning for central venous disease.

The patient's risk factors made the eventual findings clinically plausible. Central venous access devices are associated with endothelial injury, catheter-related thrombosis, and chronic stenosis [5,7]. Cancer-associated thrombosis can occur despite anticoagulation, and recurrence during reported anticoagulant use does not by itself prove nonadherence or drug failure [3,4,8]. Similar reports have described catheter-associated superior vena cava or central venous obstruction in oncology or long-term central access patients, including cases requiring endovascular treatment to restore central venous drainage [1,9,10]. This case adds a point-of-care ultrasound pitfall: the most important pathology may lie central to the routinely interrogated peripheral segments.

This case also demonstrates why the distinction between peripheral upper-extremity thrombosis and central venous thrombosis matters. Central venous obstruction can produce arm swelling, chest wall collateralization, and superior vena cava-type physiology, even when the superior vena cava itself remains patent [1,9,10]. Here, venography and intravascular ultrasound showed severe left brachiocephalic stenosis beneath acute thrombus. Aspiration thrombectomy and venoplasty improved blood flow, which suggests that both thrombotic burden and fixed central venous narrowing contributed to symptoms.

Several limitations should be acknowledged. The initial point-of-care ultrasound images and techniques were not available for review, so the exact venous segments evaluated at the bedside cannot be confirmed. The clinical record incompletely described the severity and distribution of swelling, including whether there was pitting edema, erythema, hand involvement, or visible collateral chest wall veins. CT raised concern for possible extension into the right transverse sinus, but confirmatory MR venography was not documented. Finally, because this is a single case, it should not be interpreted as evidence that all high-risk patients with negative bedside ultrasound require immediate CT; rather, it supports escalation when the negative study does not fit the anatomy, symptoms, and risk profile.

## Conclusions

In high-risk oncology patients with an indwelling port and prior catheter-associated thrombosis, unilateral arm swelling may reflect central venous thrombosis rather than isolated peripheral upper-extremity deep venous thrombosis. A negative point-of-care or peripheral ultrasound is not sufficient to rule out thrombotic disease when symptoms persist and central venous segments have not been adequately evaluated. When the clinical picture remains concerning, contrast CT, CT venography, catheter venography, intravascular ultrasound, and early specialist involvement should be considered.
